# Transitional Shock in Newly Graduated Registered Nurses From the Perspective of Self-Depletion and Impact on Cognitive Decision-Making: A Phenomenological Study

**DOI:** 10.1155/2024/6722892

**Published:** 2024-10-29

**Authors:** Zhao Yingnan, Zhang Ziqi, Wang Ting, Chen Liqin, Shi Xiaoqing, Xu Lan

**Affiliations:** ^1^Department of Nursing, The First Affiliated Hospital of Soochow University, No. 899 Pinghai Street, Suzhou, Jiangsu, China; ^2^School of Nursing, Soochow University, Suzhou, China

**Keywords:** newly graduated registered nurses, qualitative study, self-depletion theory, transition shock

## Abstract

**Background:** Newly graduated registered nurses face more challenges than their experienced counterparts, as they not only confront the high pressures of an increasingly complex medical environment but also need to quickly adapt to their jobs and role transitions. The emotional burden arising at this stage is referred to as transitional shock. Self-depletion, as proposed by Baumeister, refers to the process by which individuals exhaust their internal psychological control resources when facing challenges, subsequently affecting cognition and emotion. The occurrence of transitional shock and the process of individual self-depletion appear to be closely related. However, to our knowledge, there has been limited research exploring the occurrence of transitional shock from the perspective of self-depletion theory.

**Aim:** Investigating the emergence process of transition shock through the lens of self-depletion theory entails an examination of the mechanisms by which individuals engage in self-regulation when confronted with challenges and how transition shock manifests throughout this process.

**Designs:** A descriptive qualitative study.

**Methods:** Between August and November 2023, using maximum variation sampling and purposive sampling methods, 16 nurses were selected for semistructured interviews at a tertiary hospital in Suzhou, China.

**Results:** Employing thematic analysis, three interconnected themes were identified, encompassing the entry-level workforce challenges, the subsequent effects of energy depletion, and the sources and replenishment of energy.

**Conclusion:** As new nurses adapt to their roles and environments, they encounter numerous pressures that markedly drain their psychological energy. This ongoing depletion of self-regulation energy can lead to transitional shock, impulsive decision-making, and missed nursing care.

**Implications for Nursing Management:** Managers should implement comprehensive support strategies, including optimized work environments, enhanced training, and personal development, to help newly graduated nurses successfully transition and improve care quality and retention.

## 1. Introduction

In recent years, with the rapid development of global healthcare systems, the importance of nursing in maintaining public health has become increasingly prominent. This trend is particularly evident in China, where the role of nurses has evolved from traditional “doctor's assistants” to key members of interdisciplinary teams, encompassing multiple identities such as clinical practitioners, researchers, educators, and managers [1, 2]. While this elevation in professional positioning has brought social recognition and development opportunities, it has also placed higher demands on nurses' professional competencies. However, China's healthcare system itself faces unique challenges. Despite the existence of a public health insurance system, the proportion of out-of-pocket expenses remains high [3], leading to issues such as uneven distribution of medical resources and tense doctor–patient relationships, which impose additional stress on healthcare professionals [4]. These systemic problems, intertwined with China's distinctive cultural background, further complicate the healthcare environment. Public hospitals in China dominate healthcare service provision, and their systems are deeply influenced by Confucian philosophy, which forms the core of traditional Chinese culture and emphasizes social order, hierarchical distinctions, and collective interests [5]. This philosophy profoundly impacts various social domains in China, including the healthcare system. For instance, the Confucian concept of “hierarchical order” manifests as distinct hierarchical structures; the notion of “propriety” is reflected in the formalization and ritualization of interpersonal interactions within hospitals; and the concepts of “loyalty” and “obedience” are evident in healthcare professionals' tendency to adhere to established rules and superior directives.

In this context, newly graduated registered nurses (NGRNs) face multiple challenges. NGRNs, defined as nurses who have entered the workforce within 1 year of graduation [6], must not only adapt to the macrolevel pressures of the industry but also rapidly master microlevel professional skills. According to the 2016 regulations of the National Health and Family Planning Commission Office, NGRNs in China are required to undergo a two-year standardized training program, including rotations through 3–5 different departments. While this training system aims to comprehensively enhance NGRNs' professional competencies, it also imposes additional adaptive stress [7]. This multifaceted pressure and rapidly changing environment make NGRNs susceptible to physical and mental exhaustion and burnout. They need to adapt to new work environments, learn complex clinical skills, and manage interpersonal relationships with patients, colleagues, and superiors in a short period. This dramatic transition from student to professional nurse often triggers a series of psychological responses, most notably the phenomenon known as “transition shock” [8]. Transition shock is a complex phenomenon influenced by multiple factors, including organizational culture, work environment, and individuals [9, 10]. It not only affects NGRNs' personal development but can also lead to a series of issues such as work burnout, decreased quality of care, and high turnover rates [11–13]. Studies show that the attrition rate for newly graduated nurses after 1 year ranges from 35% to 60% [14], while in China, 20%–70% of novice nurses plan to leave their jobs within the first year of employment [15]. This not only increases hospitals' management costs but also reduces personnel allocation efficiency [16]. Considering the World Health Organization's projection of a global shortage of 5.7 million nurses by 2030 [17], effective training and retention of NGRNs are crucial for the sustainable development of the nursing workforce.

China's unique cultural background, such as the “hierarchical system and collectivism culture,” may further exacerbate the stress and challenges NGRNs face during their adaptation process. Although there have been numerous domestic and international studies on NGRNs' transition shock, these studies have primarily focused on describing the current situation and analyzing influencing factors [9, 18, 19]. Currently, there is a lack of systematic research exploring the process of NGRNs' transition shock within China's specific cultural context. This research gap not only limits our comprehensive understanding of NGRNs' transition shock but also hinders the development of effective intervention measures. The self-depletion theory provides a valuable perspective. This theory posits that individuals initially possess a certain amount of self-regulatory resources, which are consumed when performing self-regulatory tasks. When consumption reaches a certain threshold, individuals experience decreased control and a state of “fatigue.” Factors affecting the self-depletion process are diverse, including but not limited to individual factors, social support, and organizational culture [20]. In the context of NGRNs, continuous role adaptation, skill learning, and interpersonal interactions may lead to rapid depletion of self-regulatory resources. Based on this background and theoretical framework, this study employs qualitative research methods to combine the self-depletion theory with China's unique cultural and healthcare system background to present a comprehensive and China-specific process of NGRNs' transition shock. The research findings will not only provide a theoretical basis for developing intervention measures targeting transition shock among Chinese NGRNs but will also enrich the existing knowledge base in this field domestically and internationally, offering insights for cross-cultural nursing management and international nursing education.

## 2. Methods

### 2.1. Design

This study employs descriptive phenomenological as its qualitative methodology. This approach focuses on observing specific phenomena, dissecting their internal and external components, isolating key elements, and investigating the interrelations among these elements and their broader contexts [21]. Semistructured interviews were carried out with NGRNs at a tertiary hospital in the East China region from August and November 2023, aiming to capture the essence of their transitional experiences.

### 2.2. Participants

We employed purposive sampling, a method that selects participants based on their specific experiences with a particular phenomenon, enabling them to share these experiences [22]. The maximum variation sampling technique was also applied to recruit a heterogeneous sample of NGRNs across education levels, ages, hometowns [23]. Inclusion criteria are as follows: ① registered clinical nurses; ② have been working for ≤ 1 year; and ③ provided informed consent and voluntarily participated in this study. Exclusion criteria are as follows: ① nurses working in nonclinical units; ② newly graduated nurses who have not taken up their posts for various reasons (such as health, family, etc.); and ③ intern nurses.

Two researchers initiated contact with the Director of Nursing at a Class III-A hospital in the East China region, briefing them on the study's objectives and methodology. Using the roster provided by the nursing department, potential participants were identified. Ward head nurses facilitated communication with these potential participants. After detailing the study's aims and procedures, the willingness of individuals to participate was ascertained. The evaluation of the sample size is consistent, and data saturation is considered to be reached when three consecutive interviews no longer provide novel insights [24].

### 2.3. Data Collection

Based on the objectives of this study and the support of preliminary related literature, an initial interview guideline was drafted (Supporting information (available here)). Individuals with backgrounds in qualitative research, NGRNs, and self-depletion theory were organized to discuss the content of the interview guideline. After taking into account their feedback, the final interview guideline was established. Before the official research, two participants who met the inclusion and exclusion criteria were selected for a pilot interview to validate the guiding role of the interview manual. Data from the pilot interviews were then incorporated into the analysis.

Each interview was consistently conducted by a singular researcher to ensure uniformity throughout the process. Prior to commencing each interview, participants were reintroduced to the study details, and they were informed that the session would be recorded. Upon agreement, they signed an informed consent form. The questions in the interview guide were designed to be open ended and progressively structured. The conversation started with warm-up questions, such as “How have you been lately? Has your department been busy?” It then transitioned into deeper topics aligned with the study's theme, for instance, “Speaking personally, what factors do you think have led to your adaptation challenges? Can you think of a specific event as an example and elaborate on it?” Sufficient intervals were allowed between questions to provide interviewees ample opportunity to express their perspectives. In addition, anticipated follow-up questions were prepared in advance to extract more detailed and valuable information. While there existed a predefined list of interview questions, it was imperative for researchers to exhibit adaptability, modifying the sequence or depth of queries contingent on the respondent's feedback, thereby enhancing thematic exploration. All interviews were conducted in serene locations, namely, conference rooms or lounges, devoid of any external disturbances, with only the interviewer and the interviewee present. This ensured a conducive and relaxed environment, optimizing the exploration of the central themes. Each session spanned a duration ranging from 30 to 60 min. A professional recording pen was employed for audio capture. Audio recordings were transcribed by specialized personnel within the team, with the research group reviewing the transcriptions for accuracy prior to analysis.

### 2.4. Data Analysis

Data analysis was conducted using thematic analysis [25], independently carried out by two researchers experienced in qualitative studies. Initially, both researchers read and familiarized themselves with the data, striving to understand the interviewees' perspectives. Subsequently, they performed a line-by-line reading and analysis of the material, aiming to code each sentence and highlight paragraphs that were uncertain or needed discussion. To ensure the accuracy of the coding process, the following principles were applied as guidelines: (a) code for as many themes/patterns as possible; (b) retain a small portion of the data surrounding the origin of the code as excerpts; and (c) after coding, the main focus of the two researchers was to consolidate the fragmented codes into a broader thematic framework through discussion and analysis to reach a consensus on the emergent themes. The next step involved reviewing the themes, organizing all relevant codes and data excerpts under each theme and rereading them to ensure (a) coherence and significance of the codes and data within each theme without omissions and (b) clear distinctions between themes. Mind mapping was utilized to meaningfully organize the themes. Finally, themes were defined, and representative, vivid narratives were selected to compile the report. NVivo 12 software was used for data organization and analysis.

### 2.5. Rigor

Prior to the formal initiation of the study, the primary investigator and research team members conducted an extensive review of literature pertaining to transition shock among novice nurses and had published related articles, ensuring a robust research background and foundation. Regarding research design and methodology, the investigator had completed professional training in qualitative research and published multiple qualitative studies, thus ensuring the scientific rigor of the investigation. Before interviews, all equipment was thoroughly checked for proper functionality, with particular attention given to the quality of audio recording devices. To establish rapport with interviewees, initial contact was facilitated through introductions by respective department head nurses, followed by connecting via WeChat. Given that the interview process involved audio recording, preinterview communication was conducted to minimize potential defensive responses from participants, assuring them of strict confidentiality to encourage more candid responses to relevant inquiries. During interviews, questions were formulated in a neutral manner to mitigate the likelihood of participants providing socially desirable responses. Interviews were conducted in quiet, undisturbed environments, with meticulous documentation of interview content. Postinterview, verbatim transcription was performed, with researchers repeatedly reviewing audio recordings to ensure accurate documentation of interview content. Transcripts were independently coded by two researchers, followed by iterative analysis by the research group. Analysis results were repeatedly compared with the transcripts to ensure credibility. Throughout the interview process and data analysis, researchers endeavored to bracket their preconceptions and perspectives, closely aligning with participants' thoughts to authentically capture and reflect their attitudes. Quotations presented in the results section were derived directly from interviewees' original narratives, thereby ensuring the authenticity of the findings.

### 2.6. Ethical

The study was approved by the Ethics Committee of the First Affiliated Hospital of Soochow University (SDFY2023 no. 167). All participants provided verbal and written consent to participate, with only the researchers having access to the digital recordings and textual records. Reporting was informed by the Standards for Reporting Qualitative Research (SRQR) checklist [26].

## 3. Results

**Figure 1:**
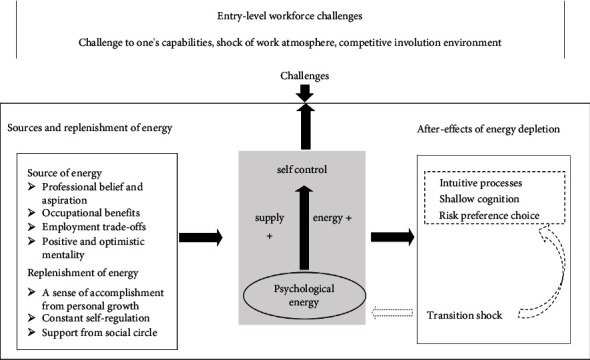
Thematic framework.

Interviews were conducted with 16 participants. Considering the maximum variation in the participants' gender, educational background, whether they interned at this hospital, and place of origin, we used purposive sampling.  Table 1 provides an overview of the general characteristics of the participants. Two researchers repeatedly read the transcribed texts to familiarize themselves with the data content. Subsequently, they coded the data. The researchers then engaged in analytical discussions, combining related codes into potential themes and reviewing the relationship between these themes and the original data. Finally, the research team defined and named the final themes, ensuring that these themes accurately reflected the overall meaning of the data (Table 2). Through this process, we identified three interrelated themes (Figure 1), grounded in the theory of self-deterioration, which revealed the sources of energy for NGRNs to cope in a challenging workplace, as well as the mechanisms through which transitional shock occurs and affects cognitive decision-making.

### 3.1. Entry-Level Workforce Challenges

Upon entering the clinical setting, new nurses face shocks including the test of personal capabilities, the work atmosphere, and the competitive environment.

#### 3.1.1. Challenge to One's Capabilities

Upon entering clinical practice, NGRNs faced challenges in three primary areas: professional knowledge and skills, handling emergency situations, and complex interpersonal communication.

##### 3.1.1.1. Professional Knowledge and Skills

*“Leaders often ask questions during morning meetings. Other than when I was preparing for postgraduate entrance exams and reading books, I hardly ever looked at them afterwards. I'm not well-versed in professional knowledge. When I can't provide an answer, even if the leader doesn't criticize me, I lose confidence. Over time, this becomes very anxiety-inducing.”* (Nurse 10)

*“My difficulty might be that my operational skills are not very good. I tend to be somewhat unfamiliar with the operational, and when I don't do well, I feel a lot of pressure.”* (Nurse 5)

##### 3.1.1.2. Handling Emergency Situations

*“In the intensive care unit, it's common to encounter emergencies, like a sudden rupture of a major blood vessel leading to immediate resuscitation, or particularly severe situations like ventricular fibrillation. I feel a sense of helplessness and get scared. When pushing the emergency cart, I run and tremble at the same time.”* (Nurse 3)

*“When you have a critically ill patient in your care, you never know how their condition might suddenly change throughout the day. This makes me very anxious before starting work each day, and the pressure is immense. It makes me not want to go to work.”* (Nurse 1)

##### 3.1.1.3. Complex Interpersonal Communication

*“The main difficulty comes from the patients. Some patients or their relatives can be truly unreasonable and noisy, quite difficult to deal with. When I encounter such situations, I get very angry. I'm quite perplexed and don't know what to do.”* (Nurse 7)

*“The difficulty lies in interpersonal relationships; it's quite challenging. I can't fit in with the team. I might communicate smoothly with people of similar years of experience, but when speaking with more senior individuals, I have to pay attention to my attitude, facial expression, and tone. I don't understand it, and they don't like me either.*” (Nurse 2)

#### 3.1.2. Shock of Work Atmosphere

In Chinese, the work atmosphere refers to the atmosphere and ambiance concerning the interrelationships among employees, communication styles, leadership styles, and work attitudes in a specific work environment [27]. In this study, the work atmospheres that influenced NGRNs included the following: the veteran's attitude toward instructing newcomers, the “we are a team” working style, different leadership paradigms, and we need a warm haven.

##### 3.1.2.1. The Veteran's Attitude Toward Instructing Newcomers

*“Some department atmospheres tend to suppress and belittle newcomers. When you are constantly in a state of being negated, it makes you anxious and at a loss, leading you to think, “Why am I so inadequate? Why can't I adapt?””* (Nurse 13)

*“Some things, they insist I do their way. What confuses me is that my approach doesn't violate any fundamental principles. I would argue with the senior staff, which often lands me in trouble. Rumors would spread in the department about how I don't listen to the advice of senior staff, leading them to form negative opinions about me. It's very frustrating.”* (Nurse 14)

##### 3.1.2.2. The “We Are a Team” Working Style

*“In some departments, people only take care of the patients in their assigned beds and bury themselves in their own work. When I actively seek help, they ignore me, making me feel isolated and unsupported. It feels like they expect me as a new nurse to do more work for them.”* (Nurse 14)

*“In our department, we basically work as a team. In this kind of team atmosphere, things are somewhat more relaxed. No matter what happens, you feel like you have the support of the entire department behind you, which eases some of the pressure.”* (Nurse 11)

##### 3.1.2.3. Different Leadership Paradigms: Democratic Leadership Style

*“I have never been anxious about exams. One time, the leader asked me how much I scored, and I told her it was somewhat low, and gave her my score. She just said, “Oh dear, our department is going to be at the bottom,” and didn't say anything else.”* (Nurse 2)

“*I've been late to this department five times, but the leadership was very understanding. They didn't scold or criticize me, but gave me some leeway, telling me just to go have my meal. I was quite touched because, in reality, being late isn't a good thing.”* (Nurse 8)

##### 3.1.2.4. Different Leadership Paradigms: Authoritarian Leadership Style

*“For instance, during the last examination I barely passed and then got scolded severely by the head nurse. I felt really down for several days after that. The head nurse has very stringent requirements, insisting on scores above 80. After being criticized, I constantly doubted myself. Being scolded naturally doesn't feel good, and not meeting her expectations meant I was frequently on the receiving end of the head nurse's reprimands.”* (Nurse 6)

*“For instance, during the morning meeting when you are asked a question and you can't answer correctly, the head nurse criticizes you harshly. It ruins your entire day, leaving you feeling frustrated, helpless, and very afraid of her.”* (Nurse 12)

*“Leaders don't care about your emotional well-being; they only focus on whether you've done your job well. They only talk to you when your performance is lacking, and these conversations feel cold and impersonal.”* (Nurse 13)

##### 3.1.2.5. We Need a Warm Haven

*“Work isn't feared for being tiring, but for having a bad atmosphere. If the atmosphere is good and the relationships are harmonious, it's okay to be a bit tired from work. However, a bad atmosphere can make you mentally exhausted and even cause you to doubt your profession.”* (Nurse 10)

*“I place greater importance on the atmosphere in the department and interpersonal relationships. I don't care much about whether the job is tiring or if the workload is heavy. The most important thing is whether or not the job brings happiness. It's okay to be a bit physically tired.”* (Nurse 8)

*“Even though the job keeps me on my toes, the colleagues in the department are great. There aren't any underhanded or competitive intentions; we focus solely on our professional tasks. They also lend a hand when needed, creating a positive working atmosphere. With such an environment, I find the motivation and enthusiasm to work.”* (Nurse 1)

#### 3.1.3. Competitive Involution Environment

Within the expansive labor landscape of China, industries across the board are marked by intense competition. Driven by factors such as the aspirations for career progression, societal anticipations, and more, the “involution” competitive dynamic has expanded beyond just the individual employees. Leadership roles and institutional entities, including hospitals, are now entrenched in this horizontal rivalry [28]. As a result, there is a heightened emphasis on elevating the professional competencies and criteria for employees.

##### 3.1.3.1. Dense and Rigorous Training and Evaluation

“*The monthly training and evaluations conducted by the nursing department bring a lot of pressure. Every time there's an examination, I have to practice many times within the department to prepare.”* (Nurse 1)

“*Additionally, there's the need to accumulate educational credits, undertake practical operation tests, and participate in nursing rounds. If there are competitions, active participation is expected*.” (Nurse 6)

*“My most significant sentiment this year is that there are just too many evaluations -truly, an overwhelming number. I initially had a passion for clinical work, but the incessant assessments have placed immense pressure on me. The added element of rankings further intensifies the stress, making the period leading up to each evaluation mentally taxing.”* (Nurse 10)

##### 3.1.3.2. The Pressure and Discrepancy Brought About by Higher Education

“*I'm not concerned about my job responsibilities; it's the research tasks that worry me. After all, I entered in the capacity of a postgraduate student, so I anticipate some research-related duties. My main concern is whether I can handle and excel in the research aspect.”* (Nurse 10)

*“Given my status as a postgraduate student, the gap between what I expected my job to entail and the reality is vast. In practice, I'm constantly engaged in clinical duties, but my interest doesn't lie there; I'm keen on dedicating more time to research. It feels like my move to the hospital has resulted in more losses than gains, as if I've relinquished my ideals. This has made me very anxious. I'm contemplating whether opportunities in smaller hospitals might be less for a postgraduate and if that would allow me more leeway for research.”*(Nurse 15)

*“The pressure might be even greater. Having spent a prolonged time in academic pursuits, one might not be thoroughly familiar with clinical practices. Moreover, entering the field with a postgraduate identity, there's the fear of underperforming and potentially facing criticism from colleagues. Hence, the stress is significant and it's quite anxiety-inducing.”* (Nurse 8)

### 3.2. After-Effects of Energy Depletion

When individuals engage in sustained self-control, psychological energy resources are depleted, leading to negative effects on subsequent self-regulatory activities, including cognitive, emotional, and behavioral issues [29]. In this study, newly graduated nurses experienced transition shock, impulsive decision-making, shallow cognition, and missed nursing care among other issues following depletion.

#### 3.2.1. Emotional Experience

##### 3.2.1.1. Transition Shock

Owing to the complexities introduced by elements such as distinct roles, environmental shifts, job expectations, specific duties, and interpersonal dynamics, participants consistently conveyed having undergone emotions emblematic of transition shock. This spectrum of emotions encompassed feelings like stress, anxiety, bewilderment, and a sense of isolation. Notably, these emotional intensities peaked between the third and sixth months, which resonate directly with the phases outlined in the transition shock theory [8].

“*These negative emotions tend to be more pronounced during the initial 3 months.*” (Nurse 1)

*“Prior to joining, I was filled with expectations. But upon starting, I was immediately engulfed in significant stress and overwhelming anxiety. This intense emotional state persisted for roughly six months, then gradually eased.”* (Nurse 15)

*“During the first three months, I was highly anxious and felt lost about this profession. The amount of adaptation and learning required was overwhelming, both in terms of the job and interpersonal relations.*” (Nurse 6)

*“When I first started, I was filled with fear, worried that I wouldn't perform well or adapt quickly since I tend to process things at a slower pace. Later on, I felt constantly agitated and under immense pressure. But there was no choice; I had to soldier on. Thankfully, in the latter half of the year, I gradually became more accustomed to my role.*” (Nurse 9)

*“During the first three months, I felt significant pressure, as this was a distinct shift from my internship, requiring me to assume responsibilities solo. As time progressed, anxiety set in, rooted in my apprehensions about potentially mismanaging patients and navigating through numerous evaluations. Thus, even the mere thought of work became a source of anxiety.”* (Nurse 5)

In this study, nearly all participants indicated that transition shock affected their state during.

#### 3.2.2. Cognition

##### 3.2.2.1. Shallow Cognition

Individuals who have undergone self-depletion rely more on superficial cognitive processing; they do not question instructions but mechanically follow them, unwilling to expend further energy to verify or reason the validity of the information [30, 31].

*“At the beginning, when you have a mentor guiding you, you're inclined to question and ask “why.” But later on, when you're in charge of patients on your own, amidst the stress and anxiety, your primary focus becomes finishing tasks hastily. In such a chaotic state, you merely aim to get things done, barely pausing to consider the rationale behind certain medical orders and their appropriateness.”* (Nurse 8)

*“Because the time in the ICU is tight and the processes are very close-knit, and being a novice, I naturally work slower, thus the pressure is high. Otherwise, I can't finish everything even by the time I'm off work. There's no thought, nor is there time for me to reflect on the reasonability of this medical order or treatment, it's just mechanistic step-by-step execution. Moreover, my specialized knowledge is also lacking; I can't reflect on much, and even if something's not reasonable, I can't discern it.”* (Nurse 3)

#### 3.2.3. Risk Preference Choice

##### 3.2.3.1. Missed Nursing Care

Individuals may fear they lack the necessary resources to handle challenges, prompting them to choose more risk-averse actions [32]. Such avoidance could directly lead to missed nursing care.

*“These emotions are distracting, making it hard to concentrate. When I'm at work, I tend to forget things here and there. I can't afford to forget too much, especially the essential tasks. I make sure to jot down important tasks in my notebook. As for the less critical tasks, if I don't write them down, I might forget to do them.”* (Nurse 1)

“*During that time, negative emotions frequently plagued my workdays, leading to occasional oversights. While these oversights were typically minor tasks, I remained vigilant about the crucial ones, fearing the repercussions of any mishaps.”* (Nurse 13)

#### 3.2.4. Intuitive Processes

##### 3.2.4.1. Impulsive Decision-Making

When individuals lack the resources for self-control, they are more inclined to rely on the intuitive processes of the dual-process model [33], resulting in impulsive decision-making.

“*Because negative emotions will affect my judgment, making it impossible for me to think more calmly, there is no room for too much thought at that moment; otherwise, I would not make some hasty and impulsive decisions.*”(Nurse 11)

“*Such emotions also affect communication with patients' families. I don't have the patience to communicate, I can't control myself, and when I'm extremely irritated, I start arguing with them. In hindsight, it was a bit impulsive.*” (Nurse 2)

### 3.3. Sources and Replenishment of Energy

Based on the analysis, it is concluded that individuals draw upon internal and external sources of psychological energy and replenishment to cope with stress, challenges, and emotional states, aiming to maintain emotional balance and mental wellbeing and to better adapt to their work [34].

#### 3.3.1. Source of Energy

##### 3.3.1.1. Professional Belief and Aspiration

“*In terms of my original intent, I genuinely appreciate this job. My initial choice of this profession might have been somewhat serendipitous, but I feel a strong sense of mission. Sometimes, when a patient is discharged, I also feel a great sense of accomplishment.*” (Nurse 3)

“*What sustains me is the anticipation for this industry and my own future.*” (Nurse 10)

“*I believe that the nursing industry has a promising future, and I have confidence in it. I aspire to be a part of that future. While I might not become a central figure, at the very least, I hope to make some contributions and achievements in this field. That's my motivation.*” (Nurse 8)

##### 3.3.1.2. Occupational Benefits

“*Sometimes the work is very tiring and the pressure is also great, but when you see the money you're earning is really not a small amount, you grit your teeth and get through it.*” (Nurse 14)

*“For the salary, I can endure a bit more. I often think that as long as I push through today, tomorrow will be a new beginning, which gradually eases the pressure.”* (Nurse 4)

“*Moreover, if I pass the exam for this hospital, the salary will be a bit higher, and it will be more convenient for my family members to see a doctor. I suppose this is my motivation to persevere.*” (Nurse 7)

##### 3.3.1.3. Employment Tradeoffs

“*When I'm particularly frustrated, I think about quitting. But then, what else can I do? Given the current economic situation, jobs are hard to come by. Compared to those without a job, I'm already in a much better position. Thinking this way provides some solace, so I just grit my teeth and push on.*” (Nurse 2)

“*I have to persist for this job opportunity. Nowadays, it's difficult to find a job in a big hospital. Compared to the predicament of being unemployed without a job, my stress and anxiety are nothing.*” (Nurse 5)

##### 3.3.1.4. Positive and Optimistic Mentality

*“Compared to the people around me, I am a bit more optimistic and open-minded. Sometimes I feel a bit of pressure and anxiety, but it's not serious and doesn't affect me much.”* (Nurse 5)

“*I feel that I have good psychological quality. Having experienced especially busy departments and departments with a poor atmosphere, I have been able to persist without considering resigning or allowing depression and anxiety to affect my life.”* (Nurse 4)

#### 3.3.2. Replenishment of Energy

##### 3.3.2.1. A Sense of Accomplishment From Personal Growth

“*Once, when I was taking a patient out for an examination and had just finished a CT scan, moving him to the transport bed, he suddenly went into ventricular fibrillation. It was my first time performing CPR, and my mind was in a haze. My colleague said I flew onto the bed to perform CPR for the patient, and in the end, we managed to resuscitate him. Experiences like this that allow me to grow give me a continuous sense of accomplishment, providing me with strength and boosting my confidence*.” (Nurse 11)

“*In my work, I set small goals for myself: In the short term, I don't demand that this paper must be accepted, but I want to submit it. Achieving a small goal, and continuously reaching targets, gives me a sense of solidity and makes me feel more confident and passionate about my work state*.” (Nurse 10)

##### 3.3.2.2. Constant Self-Regulation

*“Most of the time, I manage on my own. I don't really share with my colleagues. After all, colleagues are colleagues, and saying too much can still bring about social pressure.*” (Nurse 11)

*“I'm from another province and city, so I don't have many friends here, only colleagues. There's no one to comfort me, so I can only distract myself by watching TV series and browsing TikTok.*” (Nurse 12)

*“Complaining to friends or colleagues might become a topic of gossip for others, so it's better to regulate my emotions by myself. After shopping, I feel motivated to work and earn money again.*” (Nurse 4)

“*At that time, I was aware that my mental state might not be right. I tried to self-regulate, but the results might not have been particularly good*.” (Nurse 8)

##### 3.3.2.3. Support From Social Circle

“*When I'm feeling particularly down during certain times, I'd talk to my friends about it. Sometimes, I'd also go shopping, or scroll through my phone and watch short videos, which tends to improve my mood significantly.*” (Nurse 3)

“*When I'm under a lot of stress, I'll go cycling with friends, and having them listen to me talk is also a way to relieve pressure*.” (Nurse 9)

“*When negative emotions become too overwhelming, I feel like I can't handle it without some adjustment. So I'll take advantage of my days off to hang out or travel with friends, and I feel much better after returning.*” (Nurse 5)

## 4. Discussion

This research delves into the occurrence of transition shock under the self-depletion theory framework. The participants outlined three interconnected themes: entry-level workforce challenges, the subsequent effects of energy depletion, and the sources and replenishment of energy. These themes uncover a sequential phenomenon, where NGRNs confront diverse challenges at the onset of their careers, managing these challenges through their psychological energy resources. This ongoing depletion of psychological resources subsequently influences their emotional and cognitive decision-making processes.

NGRNs encounter a multitude of challenges at both individual and environmental levels. Individually, they face challenges in aspects such as interpersonal relationships, responsibilities, knowledge, and skills aligning with the themes identified in this study [18, 35, 36]. Regarding the workplace atmosphere, the majority of prior studies indicate that the implementation of a mentorship system can mitigate the transition shock experienced by NGRNs [9]. However, limited research has examined how the pedagogical approach of mentors also affects the transition experience of NGRNs. E.W. Taylor in his theory of adult learning highlighted that transformative learning for adults is a process encompassing reflection and long-term practice, as well as a complex interplay of thoughts and emotions, based on a relationship of mutual dependency built on trust [37]. China's healthcare service system increasingly demands nurses with critical thinking skills, proactive learning abilities, and adaptability. However, Chinese teaching philosophies and student-teacher relationships are deeply influenced by Confucian culture [38]. The student–teacher relationship is relatively formal with clear hierarchical distinctions, where students are expected to respect and obey teachers. Typically, the teaching style emphasizes teacher-led instruction with passive student reception. Evidently, this educational approach is unsuitable for training novice nurses. Traditional Chinese culture, particularly Confucian philosophy, has shaped educational practices and value systems, influencing both the content and methods of nursing education while also permeating the organizational structure and operational modes of the healthcare system. This multifaceted influence has resulted in communication gaps or cultural misalignments between the educational approach for nurses and the operational realities of healthcare services. For instance, traditional hierarchical concepts manifest as strict student-teacher relationships in the educational system and translate into explicit rank structures in the healthcare system, potentially hindering novice nurses' adaptation to teamwork and equal communication requirements. Confucian culture's emphasis on experiential inheritance and respect for authority is reflected in teacher-centered knowledge transmission models in educational culture. In the healthcare system, experienced medical professionals are often viewed as unquestionable authorities. This may lead to novice nurses overly relying on guidance from senior colleagues, lacking independent thinking and innovative capabilities. Understanding the complexity of these intertwined cultural elements is crucial for improving the training and adaptation of novice nurses. While respecting traditional culture, we need to actively reform educational methods. This includes introducing more case analyses and scenario simulations, encouraging active student participation, enhancing clinical mentor training, and creating a more open and egalitarian environment for medical communication. These measures aim to better adapt to the demands of modern medical practice, mitigate the challenges posed by cultural intersections, and provide improved transitional support for novice nurses. In terms of the professional environment, the recent trend of “involution” in China's workplace and educational sectors has been intensifying. The concept of “involution” was first proposed by American anthropologist Clifford Geertz [39]. Its features include irrational blind following, formalized passive participation, and innovation-less overcompetition. The public continuously strives to improve themselves to compete for resources, thereby raising the threshold for resource competition and entering a state of “irrational competition.” This phenomenon may lead to increased work difficulty, heightened individual psychological stress, reduced self-identification, and public anxiety [40]. In addition, as highly educated members of hospital nursing teams, nursing graduate students bear higher expectations from leaders and colleagues. They are compelled to participate in “involution,” and, in addition to this, must complete assessments and training equivalent to undergraduate and diploma-level nurses. Therefore, they might experience higher levels of transitional shock [41].

Facing the challenges outlined, according to the self-depletion theory, individuals initiate self-regulation using their own resources, and the depletion process impacts emotions, cognition, and decision-making [42]. New nurses, being in a sensitive and emotionally unstable state during their transitional period [43], commonly experience varying degrees of transitional shock [44–46]. This study's participants reported significant experiences within the first 3 months of work, aligning with the transitional shock theory [8].

However, they also noted that transitional shock adversely affected their cognitive decision-making, including superficial cognition, impulsive decisions, and missed nursing care. Research by Labrague and Santos [13] further confirms that transitional shock in new nurses can lead to missed nursing care and incidents of errors. This discrepancy with the self-depletion theory may partly arise because the negative emotions triggered by emotional depletion themselves consume a portion of an individual's limited energy for processing, leaving fewer resources for self-regulation and thus impacting cognitive decision-making. In addition, individuals may be more perceptive to changes in emotions compared to cognitive decision-making, leading participants to subjectively attribute the impact on cognitive decision-making to emotional changes. Consequently, further quantitative research is needed to validate whether transitional shock affects cognitive decision-making and thereby leads to adverse outcomes. To mitigate the effects of energy depletion, efforts should not only focus on reducing depletion but also on enhancing the self-control energy of NGRNs.

The self-control theory conceptualizes self-control as a finite resource that determines an individual's self-regulation capacity [47, 48]. Research has demonstrated that this resource can be both replenished and fortified [49]. First, self-control resources can be restored to their initial level following periods of rest. Second, the energy depleted from self-regulatory efforts is dynamic and restorable, similar to how muscle strength can be built through exercise. Third, individuals exhibit variability in their self-regulation capabilities, with the total quantity of self-control resources largely depending on the individual [50]. Assisting NGRNs in enhancing and rapidly recovering their self-control resources is crucial for minimizing occurrences of self-depletion. Future interventions could thus be designed with an emphasis on this crucial aspect, adapting strategies to fit the cultural nuances of various countries and regions. This study's participants emphasized their aspirations for career advancement and job security as primary psychological resources. Under the influence of Confucian culture and the educational system, employment perspectives in China, which favor job stability and income, differ from those in the West. Despite the relatively modest income levels within the nursing profession in China, the sector is perceived to offer comparative advantages in terms of job availability and stability, which are key reasons for individuals choosing and committing to this career path. In addition, the pursuit of career progression is not only a short-term motivator for new nurses but also constitutes critical psychological support for the stable and sustainable growth of their professional lives [51]. Nevertheless, career development paths for nurses in China are limited. To address this, management should endeavor to expand these paths, providing more than just additional educational and training opportunities. This includes offering more comprehensive professional guidance, career planning, introducing extensive international certification systems, and improving nurses' social recognition at the societal level. Psychological interventions in the future should not be limited to conventional group counseling and positive psychological interventions like mindfulness [52] but should also introduce NGRNs to self-regulation techniques that can expedite the recovery of psychological energy, such as emotional management, positive thinking, and coping strategies, effectively improving their self-regulation abilities. Moreover, it is important for managers to help nurses develop a sense of professional achievement and confidence, bolstering their psychological resilience. Feeling satisfied and motivated by each success and progression not only contributes to personal career advancement but also to the enhancement of mental health and quality of life [53, 54].

This study has several limitations. First, it was conducted in only one hospital in the East China, thus limiting the generalizability of the findings. Second, the sample size was small, but the study's use of maximal variation purposive sampling ensured data richness. The research team agreed, after internal discussion, that data saturation was achieved as no new codes were generated in subsequent interviews, suggesting that the sample size was sufficient to meet the study objectives. Future studies could extend to broader regions and countries in a multicenter approach to address these limitations.

## 5. Conclusion

Given the global shortage of professional nurses, the transition of NGRNs is a crucial issue for hospitals and nursing managers. In this study, grounded in the framework of self-depletion theory, we conducted an in-depth analysis of the challenges encountered by NGRNs during their professional transition, the depletion and recovery of their self-regulatory energy, and the subsequent effects of energy depletion. The findings revealed that NGRNs face multiple pressures while adapting to their professional roles and work environments, significantly draining their psychological energy. The continuous depletion of self-regulatory energy may lead to transition shock, impulsive decision-making, and missed nursing care among NGRNs. Future research and intervention should consider ways to enhance and rapidly replenish their self-control energy. These insights provide crucial theoretical and practical guidance for facilitating the smooth transition of NGRNs into their professional roles.

### 5.1. Implications for Nursing Management

The self-depletion theory perspective can enhance our understanding of the transition phenomenon among newly graduated nurses, thereby facilitating the development of targeted preventive measures and the assessment of their effectiveness. First, given the multiple stressors and psychological energy depletion faced by newly graduated nurses, it is recommended to establish a multitiered support system comprising experienced nurses, psychological counselors, and managers. This system should provide stress management training and self-regulation skill workshops to help NGRNs quickly recover and enhance their self-control capabilities. Second, regarding transitional challenges, a stratified transitional support program should be established: designing targeted support plans based on the different stage-specific needs of NGRNs. Third, to address potential nursing negligence resulting from continuous depletion of self-regulatory energy, managers should optimize work allocation and shift arrangements, implementing a “stepped” shift system that gradually increases work complexity and responsibility. Finally, considering China's unique cultural background, managers should focus on harmonizing traditional cultural values with the demands of modern nursing practice, creating a work environment that both respects tradition and encourages innovation to help newly graduated nurses better adapt to their professional transition.

## Figures and Tables

**Table 1:** Participant characteristics.

Characteristic	*N*
Sex	
Females	12
Males	4
Age (years)	
≤ 21	1
22–25	11
> 25	4
Education	
Junior college	9
Undergraduate	4
Master's degree or above	3
Hometown	
Local	7
Out of town	9
Monthly income (¥)	
≤ 5000	0
5000–10,000	16
> 10,000	0
Whether to choose the nursing profession voluntarily	
Yes	10
No	6
Current rotation department	
Internal medicine	4
Surgery	6
Emergency department	3
ICU	3
The internship hospital and the employment hospital are the same	
Yes	5
No	7
Frequency of night shifts	
0 times a week	3
Once a week	13
Frequency of overtime work	
Never worked overtime	1
Occasionally work overtime	14
Often working overtime	1
Personal personality type	
Type of introversion	7
extroverted	9

**Table 2:** The subthemes, codes, and representative participant quotes under each theme.

Theme	Subtheme	Code	A typical instance
Challenges of entering the workforce	Challenge to one's capabilities	Professional knowledge and skills	“My difficulty might be that my operational skills are not very good. I tend to be somewhat unfamiliar with the operational, and when I do not do well, I feel a lot of pressure.” (Nurse 5)
		Handling emergency situations	“When you have a critically ill patient in your care, you never know how their condition might suddenly change throughout the day. This makes me very anxious before starting work each day, and the pressure is immense. It makes me not want to go to work.” (Nurse 1)
		Complex interpersonal communication	“The main difficulty comes from the patients. Some patients or their relatives can be truly unreasonable and noisy, quite difficult to deal with. When I encounter such situations, I get very angry. I am quite perplexed and do not know what to do.” (Nurse 7)
	Shock of work atmosphere	The veteran's attitude towards instructing newcomers	“Some department atmospheres tend to suppress and belittle newcomers. When you are constantly in a state of being negated, it makes you anxious and at a loss, leading you to think, “why am I so inadequate? Why cannot I adapt?” (Nurse 13)”
		The “we are a team” working style	“In our department, we basically work as a team. In this kind of team atmosphere, things are somewhat more relaxed. No matter what happens, you feel like you have the support of the entire department behind you, which eases some of the pressure.” (Nurse 11)
		Different leadership paradigms	“For instance, during the morning meeting when you are asked a question and you cannot answer correctly, the head nurse criticizes you harshly. It ruins your entire day, leaving you feeling frustrated, helpless, and very afraid of her.” (Nurse 12)
		“We need a warm haven”	“Work is not feared for being tiring but for having a bad atmosphere. If the atmosphere is good and the relationships are harmonious, it is okay to be a bit tired from work. However, a bad atmosphere can make you mentally exhausted and even cause you to doubt your profession.” (Nurse 10)
	Competitive involution environment	Dense and rigorous training and evaluation	“The monthly training and evaluations conducted by the nursing department bring a lot of pressure. Every time there is an examination, I have to practice many times within the department to prepare.” (Nurse 1)
		The pressure and discrepancy brought about by higher education	“I am not concerned about my job responsibilities; it is the research tasks that worry me. After all, I entered in the capacity of a postgraduate student, so I anticipate some research-related duties. My main concern is whether I can handle and excel in the research aspect.” (Nurse 10)

After-effects of energy depletion	Emotional experience	Transition shock	“During the first 3 months, I was highly anxious and felt lost about this profession. The amount of adaptation and learning required was overwhelming, both in terms of the job and interpersonal relations.” (Nurse 6)
	Risk preference choice	Missed nursing care	“During that time, negative emotions frequently plagued my workdays, leading to occasional oversights. While these oversights were typically minor tasks, I remained vigilant about the crucial ones, fearing the repercussions of any mishaps.” (Nurse 13)
	Intuitive processes	Impulsive decision-making system	“Because negative emotions will affect my judgment, making it impossible for me to think more calmly, there is no room for too much thought at that moment; otherwise, I would not make some hasty and impulsive decisions.” (Nurse 11)
	Cognition	Superficial cognitive processing	“At the beginning, when you have a mentor guiding you, you are inclined to question and ask “why.” But later on, when you are in charge of patients on your own, amidst the stress and anxiety, your primary focus becomes finishing tasks hastily. In such a chaotic state, you merely aim to get things done, barely pausing to consider the rationale behind certain medical orders and their appropriateness.” (Nurse 8)

Sources and replenishment of energy	Source of energy	Professional belief and aspiration	“What sustains me is the anticipation for this industry and my own future.” (Nurse 10)
		Occupational benefits	“Moreover, if I pass the exam for this hospital, the salary will be a bit higher, and it will be more convenient for my family members to see a doctor. I suppose this is my motivation to persevere.” (Nurse 7)
		Employment tradeoffs	“I have to persist for this job opportunity. Nowadays, it is difficult to find a job in a big hospital. Compared to the predicament of being unemployed without a job, my stress and anxiety are nothing.” (Nurse 5)
		Positive and optimistic mentality	“Compared to the people around me, I am a bit more optimistic and open-minded. Sometimes I feel a bit of pressure and anxiety, but it is not serious and does not affect me much.” (Nurse 5)
	Replenishment of energy	A sense of accomplishment from personal growth	“In my work, I set small goals for myself. In the short term, I do not demand that this paper must be accepted, but I want to submit it. Achieving a small goal, and continuously reaching targets, gives me a sense of solidity and makes me feel more confident and passionate about my work state.” (Nurse 10)
		Constant self-regulation	“Most of the time, I manage on my own. I do not really share with my colleagues. After all, colleagues are colleagues and saying too much can still bring about social pressure.” (Nurse 11)
		Support from social circle	“When I am under a lot of stress, I will go cycling with friends and having them listen to me talk is also a way to relieve pressure.” (Nurse 9)

## Data Availability

The data that support the findings of this study are available on request from the corresponding author. The data are not publicly available due to privacy or ethical restrictions.

## Supporting Information

The interview outline can be found in the annex to the supporting information.
