# Supplementary material for: Transitional Shock in Newly Graduated Registered Nurses From the Perspective of Self-Depletion and Impact on Cognitive Decision-Making: A Phenomenological Study
Source: J Nurs Manag. 2024 Oct 29;2024:6722892. doi: 10.1155/2024/6722892 (PMC11918504; doi:10.1155/2024/6722892)
Supplement: Supporting Information — Additional supporting information can be found online in the Supporting Information section. [file 6722892.f1.docx]

**Qualitative Interview Guide**

Hello, we are currently conducting a hospital-wide survey research. The aim is to investigate the transition and adaptation of NGRNs during their first year of work, including your experiences this year and factors that have affected your adaptation difficulties. This will enable us to subsequently implement measures to facilitate your quick adaptation to clinical work and reduce negative emotions such as psychological anxiety and stress. This study has been approved by the Ethics Committee of the First Affiliated Hospital of Soochow University. The study employs purposive sampling to conduct semi-structured in-depth interviews with nurses who have worked for less than a year. The interview will take approximately 30-60 minutes and will be audio-recorded. To protect your privacy, we will not record your name and guarantee that all interview content will be kept strictly confidential, used only for scientific research, and will not lead to any leak of information from your interview process. During the interview, you have the right to refuse or withdraw at any time without any adverse effects on you.

1. Warm-up question: A year has passed in a flash. Can you still recall the scene when you first joined the hospital last year? What was it like?
2. Looking back on your work this past year, how do you feel about your overall adaptation process from student to nurse? Are there any memorable experiences? Have you gained anything from this process?
3. What difficulties have you encountered this year? Can you give an example?
4. From your perspective, what events or factors during this year have caused you to experience negative emotions at work? Can you recall and describe the specific circumstances at that time?
5. Do you think these negative emotions unconsciously affect your work? Can you give a specific example?
6. Do you actively adopt methods to help yourself adapt to work and alleviate negative emotions such as stress and anxiety? Can you give an example?
7. Have you adapted now? What supports you in resisting the unpleasantness and maladjustment during the transition process?
8. How do you think the hospital management and nursing department should help you get through this challenging year and alleviate your anxiety and maladjustment?
9. Is there anything else you'd like to say that we haven't mentioned?
